# Supplementary material for: COZOID: contact zone identifier for visual analysis of protein-protein interactions
Source: BMC Bioinformatics. 2018 Apr 6;19:125. doi: 10.1186/s12859-018-2113-6 (PMC5889581; doi:10.1186/s12859-018-2113-6)
Supplement: Supplementary file 2 — Software build. Executable binary file of the software tool. (ZIP 118,784 kb) [file 12859_2018_2113_MOESM2_ESM.zip › cozoid/java/docs/beansbinding-1.2.1-doc.zip!/org/jdesktop/el/impl/lang/ELArithmetic.html]

ELArithmetic (Beans Binding)


---


|  |  |  |
| --- | --- | --- |
| **Overview** \| **Package** \| **Class** \| **Use** \| **Tree** \| **Deprecated** \| **Index** \| **Help** | |  |
| PREV CLASS   **NEXT CLASS** | **FRAMES**    **NO FRAMES**     **All Classes** |  |
| SUMMARY: NESTED \| FIELD \| CONSTR \| METHOD | DETAIL: FIELD \| CONSTR \| METHOD |  |


---


## org.jdesktop.el.impl.lang Class ELArithmetic

```
java.lang.Object
  org.jdesktop.el.impl.lang.ELArithmetic
```

**Direct Known Subclasses:**: ELArithmetic.BigDecimalDelegate, ELArithmetic.BigIntegerDelegate, ELArithmetic.DoubleDelegate, ELArithmetic.LongDelegate

---

``` public abstract class ELArithmetic extends java.lang.Object ```

A helper class of Arithmetic defined by the EL Specification

---

| **Nested Class Summary** | |
| --- | --- |
| `static class` | `ELArithmetic.BigDecimalDelegate` |
| `static class` | `ELArithmetic.BigIntegerDelegate` |
| `static class` | `ELArithmetic.DoubleDelegate` |
| `static class` | `ELArithmetic.LongDelegate` |


| **Field Summary** | |
| --- | --- |
| `static ELArithmetic.BigDecimalDelegate` | `BIGDECIMAL` |
| `static ELArithmetic.BigIntegerDelegate` | `BIGINTEGER` |
| `static ELArithmetic.DoubleDelegate` | `DOUBLE` |
| `static ELArithmetic.LongDelegate` | `LONG` |


| **Constructor Summary** | |
| --- | --- |
| `protected` | `ELArithmetic()` |


| **Method Summary** | |
| --- | --- |
| `protected abstract  java.lang.Number` | `add(java.lang.Number num0, java.lang.Number num1)` |
| `static java.lang.Number` | `add(java.lang.Object obj0, java.lang.Object obj1)` |
| `protected abstract  java.lang.Number` | `coerce(java.lang.Number num)` |
| `protected  java.lang.Number` | `coerce(java.lang.Object obj)` |
| `protected abstract  java.lang.Number` | `coerce(java.lang.String str)` |
| `protected abstract  java.lang.Number` | `divide(java.lang.Number num0, java.lang.Number num1)` |
| `static java.lang.Number` | `divide(java.lang.Object obj0, java.lang.Object obj1)` |
| `static boolean` | `isNumber(java.lang.Object obj)` |
| `static boolean` | `isNumberType(java.lang.Class type)` |
| `protected abstract  boolean` | `matches(java.lang.Object obj0, java.lang.Object obj1)` |
| `protected abstract  java.lang.Number` | `mod(java.lang.Number num0, java.lang.Number num1)` |
| `static java.lang.Number` | `mod(java.lang.Object obj0, java.lang.Object obj1)` |
| `protected abstract  java.lang.Number` | `multiply(java.lang.Number num0, java.lang.Number num1)` |
| `static java.lang.Number` | `multiply(java.lang.Object obj0, java.lang.Object obj1)` |
| `protected abstract  java.lang.Number` | `subtract(java.lang.Number num0, java.lang.Number num1)` |
| `static java.lang.Number` | `subtract(java.lang.Object obj0, java.lang.Object obj1)` |

**Methods inherited from class java.lang.Object**

`clone, equals, finalize, getClass, hashCode, notify, notifyAll, toString, wait, wait, wait`

**Field Detail**

### BIGDECIMAL

```
public static final ELArithmetic.BigDecimalDelegate BIGDECIMAL
```

---


### BIGINTEGER

```
public static final ELArithmetic.BigIntegerDelegate BIGINTEGER
```

---


### DOUBLE

```
public static final ELArithmetic.DoubleDelegate DOUBLE
```

---


### LONG

```
public static final ELArithmetic.LongDelegate LONG
```


**Constructor Detail**

### ELArithmetic

```
protected ELArithmetic()
```


**Method Detail**

### add

```
public static final java.lang.Number add(java.lang.Object obj0,
                                         java.lang.Object obj1)
```

---


### mod

```
public static final java.lang.Number mod(java.lang.Object obj0,
                                         java.lang.Object obj1)
```

---


### subtract

```
public static final java.lang.Number subtract(java.lang.Object obj0,
                                              java.lang.Object obj1)
```

---


### divide

```
public static final java.lang.Number divide(java.lang.Object obj0,
                                            java.lang.Object obj1)
```

---


### multiply

```
public static final java.lang.Number multiply(java.lang.Object obj0,
                                              java.lang.Object obj1)
```

---


### isNumber

```
public static final boolean isNumber(java.lang.Object obj)
```

---


### isNumberType

```
public static final boolean isNumberType(java.lang.Class type)
```

---


### add

```
protected abstract java.lang.Number add(java.lang.Number num0,
                                        java.lang.Number num1)
```

---


### multiply

```
protected abstract java.lang.Number multiply(java.lang.Number num0,
                                             java.lang.Number num1)
```

---


### subtract

```
protected abstract java.lang.Number subtract(java.lang.Number num0,
                                             java.lang.Number num1)
```

---


### mod

```
protected abstract java.lang.Number mod(java.lang.Number num0,
                                        java.lang.Number num1)
```

---


### coerce

```
protected abstract java.lang.Number coerce(java.lang.Number num)
```

---


### coerce

```
protected final java.lang.Number coerce(java.lang.Object obj)
```

---


### coerce

```
protected abstract java.lang.Number coerce(java.lang.String str)
```

---


### divide

```
protected abstract java.lang.Number divide(java.lang.Number num0,
                                           java.lang.Number num1)
```

---


### matches

```
protected abstract boolean matches(java.lang.Object obj0,
                                   java.lang.Object obj1)
```


---


|  |  |  |
| --- | --- | --- |
| **Overview** \| **Package** \| **Class** \| **Use** \| **Tree** \| **Deprecated** \| **Index** \| **Help** | |  |
| PREV CLASS   **NEXT CLASS** | **FRAMES**    **NO FRAMES**     **All Classes** |  |
| SUMMARY: NESTED \| FIELD \| CONSTR \| METHOD | DETAIL: FIELD \| CONSTR \| METHOD |  |


---
